# Of periauricular pits and sinuses: understanding the masqueraders

**DOI:** 10.1590/S1679-45082014AI3020

**Published:** 2014

**Authors:** Mainak Dutta, Soumya Ghatak, Rahul Sarkar

**Affiliations:** 1 Department of Otorhinolaryngology and Head-Neck Surgery, Medical College and Hospital, West Bengal, India; 2 Department of Otorhinolaryngology and Head-Neck Surgery, R G Kar Medical College and Hospital, West Bengal, India

An eight-year-old girl presented with complaints of intermittent discharge from a pit situated in the medial surface of right pinna since early childhood. The discharge was scanty, foul-smelling and mucoid, and occasionally purulent. The frequency had increased in the last year with development of a swelling just below the pit that often got inflamed and caused pain. On examination, an opening was noted just above the lobule leading to a swelling that reached the post-auricular sulcus (Figure 1). An area of scarring and pigmentation in the lateral surface of the pinna in immediate vicinity to the external auditory canal (EAC) – reminiscent of past inflammations involving the EAC skin, could be seen that corresponded to the sinus tract (Figure 2A). Also, similar pits, which were asymptomatic were noted on the ascending helix of the right (Figure 2A) as well as left pinna (Figure 2B). After controlling the infection with antibiotics, a sinogram was ordered to assess the post-auricular lesion, which showed a sinus tract that ballooned in to the soft tissue of the neck, just below the mastoid process and EAC, and posterior to the ramus of the mandible (Figure 3). The tract was excised under general anesthesia. Peroperatively, it was found to end blindly stopping just short of invading the parotid tissue.

**Figure 1:**
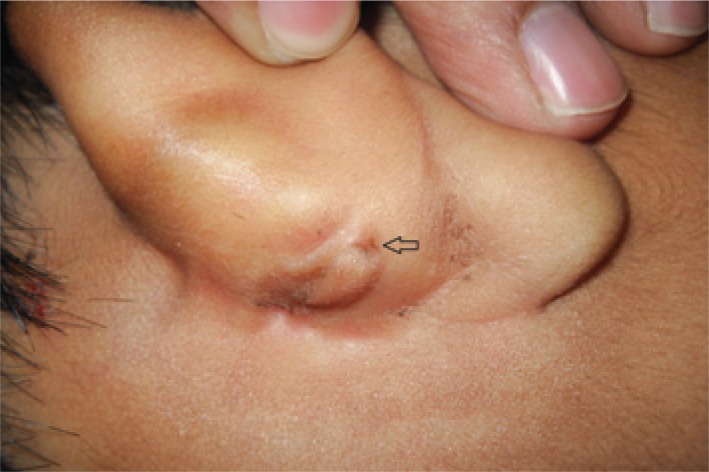
A pit (arrow) leading to a cystic cavity reaching the post-auricular groove could be seen here, situated in the lower part of the medial surface of right pinna just superior to the lobule

**Figure 2:**
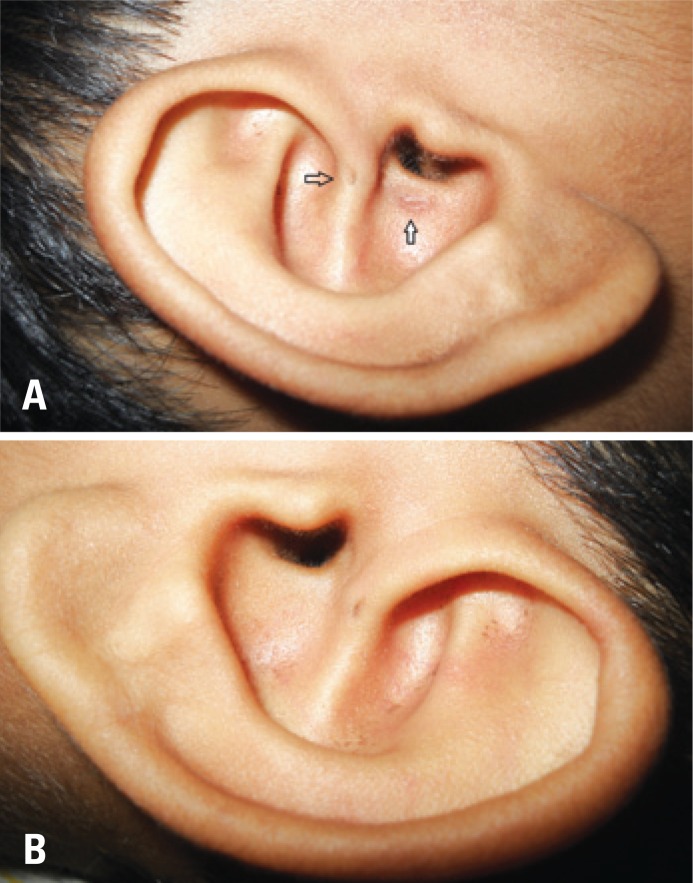
(A) The lateral aspect of the right pinna showing another pit in the ascending helix (transparent arrow). In immediate contiguity to the membranous external auditory canal is the pigmented scar (white solid arrow) that corresponds to the sinus cavity on the medial surface. (B) Intra-auricular sinus and its pit can be seen in the ascending helix of the left pinna, in an identical position with the pit in the fellow ear

**Figure 3:**
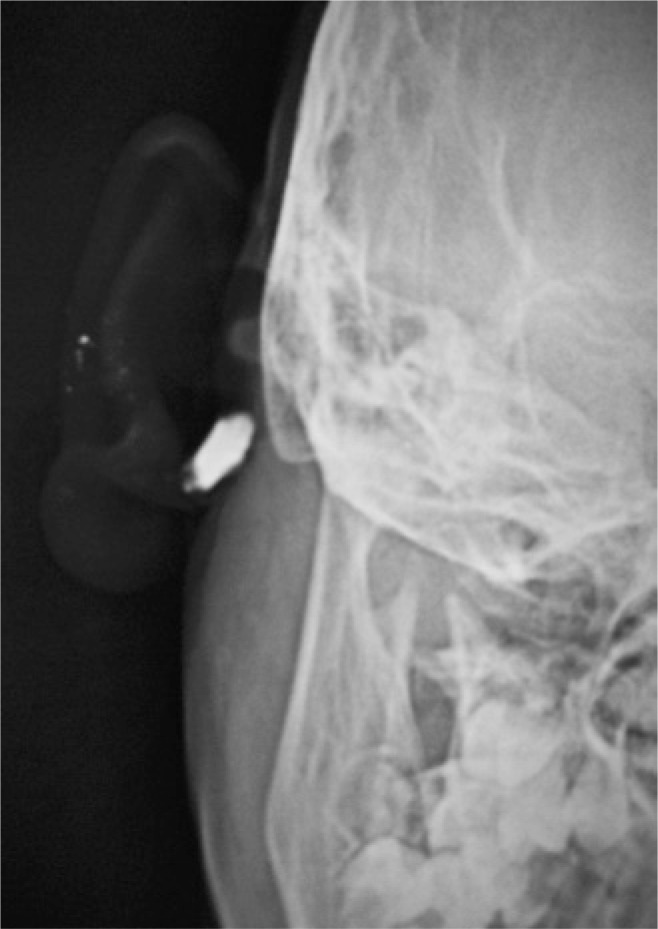
Sinogram showing ballooned sinus tract that ends blindly in front and below the right mastoid process in the soft tissue posterior to the ramus of the mandible and inferior to the external auditory canal, in the region of the anatomic location of the parotid gland

Peri-auricular pits are congenital stigmata commonly encountered by clinicians when they become infected and symptomatic. They might lead to sinuses, fistulae, cysts, or simply end in blind tracts, and are mostly noted in the pre-auricular region^(1)^ (above the tragus, in the region of incisura terminalis), but might be intraauricular^(2)^ or post-auricular. However, the clinical and embryologic implications could be different depending on their location and composition. The pinna is formed by the union of six cartilaginous hillocks (of His) derived from the first two branchial arches. The antitragus, antihelix, lobule and lower portion of helix are formed from the second arch, while the rest of the pinna is derived from the first. Pre-auricular and intra-auricular sinuses/cysts are manifestations of faulty union of the two branchial arch derivatives; they are sort of inclusion cysts/tracts and are innocuous.^(1)^ However, clinically identical cysts, sinuses or tracts may be seen in the posterior aspect of pinna and upper neck whose origin cannot be explained by the inclusion or entrapped tissue theory. In truth, they are duplications of the EAC, and are derived from malformations of the first branchial sinus which contributes to the development of the EAC.^(3)^ These may either be lined by epithelium (type I) or formed by both ectodermal and mesodermal components (type II),^(4)^ thereby often draining in the EAC or in the neck, and are in close proximity to the parotid gland and facial nerve.^(1)^ These make them vulnerable during surgical exploration. This particular child coincidentally had both branchial arch and cleft anomaly clinically presenting as pits in close anatomic proximity. It becomes very difficult in such cases to differentiate an inclusion cyst/sinus from a type I first branchial cleft anomaly which is in intimate relation with the membranous EAC.^(5)^ The situation might be misleading, and congenital syndromic disorders^(1)^ and the involvement of vital structures,^(1,5)^ both of which are known associations with branchial cleft malformations, would be missed if they are erroneously mistaken for the relatively benign and embryologically distinct preauricular and intra-auricular sinuses. Imaging (sinogram) does contribute, but the differentiation truly lies in the knowledge of the regional embryology and per-operative findings.

## References

[1] Graney DO, Sie KC, Flint PW, Haughey PH, Lund VJ, Niparko JK, Richardson MA, Robins KT (2010). Anatomy and developmental embryology of the neck. Cummings otolaryngology head & neck surgery.

[2] Marom T, Goldfarb A, Roth Y (2013). Intra-auricular sinus: first description of a variant of the pre-auricular cyst. J Craniofac Surg.

[3] Aronsohn RS, Batsakis JG, Rice DH, Work WP (1976). Anomalies of the first branchial cleft. Arch Otolaryngol.

[4] Work WP (1972). Newer concepts of first branchial cleft defects. Laryngoscope.

[5] Gonzalez-Perez LM, Prats-Golczer VE, Montes Carmona JF, Heurtebise Saavedra JM (2014). Bilateral first branchial cleft anomaly with evidence of a genetic aetiology. Int J Oral Maxillofac Surg.

